# A Novel Nonsense *MMP21* Variant Causes Dextrocardia and Congenital Heart Disease in a Han Chinese Patient

**DOI:** 10.3389/fcvm.2020.582350

**Published:** 2020-11-09

**Authors:** Zhuang-Zhuang Yuan, Liang-Liang Fan, Zi-Chen Jiang, Yi-Feng Yang, Zhi-Ping Tan

**Affiliations:** ^1^Clinical Center for Gene Diagnosis and Therapy, Department of Cardiovascular Surgery, The Second Xiangya Hospital of Central South University, Changsha, China; ^2^Department of Cell Biology, School of Life Sciences, Central South University, Changsha, China; ^3^Hunan Key Laboratory of Animal Models for Human Diseases, School of Life Sciences, Central South University, Changsha, China; ^4^University of California, San Diego, San Diego, CA, United States

**Keywords:** congenital heart defect (CHD), whole exome sequencing, stopgain variant, dextrocardia, MMP21

## Abstract

The position and morphology of human internal organs are asymmetrically distributed along the left–right axis. Aberrant left–right patterning in the developing embryo can lead to a series of congenital laterality defects, such as dextrocardia and heterotaxy syndrome. Laterality defects are a genetic condition; however, pathogenic genetic lesions are found in only one-fifth of patients. In this study, whole-exome sequencing was conducted for 78 patients with laterality defects. We identified a novel stopgain variant in *MMP21* (c.G496T; p.G166^*^) in a Chinese patient with mirror-image dextrocardia. This variant caused a truncated *MMP21* mRNA containing only the signal peptide and propeptide, while the coding sequence of matrix metalloproteinase-21 was almost entirely absent. To the best of our knowledge, this novel variant is the first homozygous stopgain variant identified in dextrocardia patients, and the first *MMP21* variant found in East Asia. Our findings expand the spectrum of *MMP21* variants and provide support for the critical role of *MMP21* during left–right patterning in the Han Chinese population.

## Introduction

In mammals, the proper left–right (L–R) patterning of internal organs is extremely complex and highly precise. Disordered L–R patterning can lead to a broad spectrum of laterality defects, including situs inversus totalis (SIT), a mirror image of situs solitus (SS), and heterotaxy (HTX), in which at least one organ is discordant along the left–right axis (1, 2). Dextrocardia, a rare congenital heart anomaly and HTX, is caused by the failure of normal L–R asymmetry patterning during heart development. As one of the first congenital heart malformations to be recognized, dextrocardia was mentioned in the early 17th century (3). SIT is rarely associated with congenital malformations, while HTX is highly associated with a series of congenital malformations. Most dextrocardia patients have other defects of the heart and abdominal organs (4).

Matrix metallopeptidase 21 (*MMP21*) encodes a member of the matrix metalloproteinase (Mmps) family that is known to hydrolyze extracellular matrix (ECM) components and is crucial for morphogenesis (5, 6). Vertebrates Mmps and their inhibitors are expressed in cardiomyocytes during the early stages of cardiac development, which are required for early heart tube assembly and modulate cardiac morphogenesis events, such as heart tube formation, heart directional looping, and differentiation of ostial cells (5).

Using N-ethyl-N-nitrosourea (ENU)-induced mutagenesis and whole-exome sequencing (WES), Li et al. (7) and Akawi et al. (8) identified *Mmp21* mutations in mice with congenital heart disease (CHD) and laterality defects, respectively (7). Additionally, *MMP21* mutations in several HTX families and sporadic cases were identified by WES (7–11). These mutations and laterality phenotypes in humans and mice indicated that *MMP21* plays a critical role in the L–R patterning of visceral organs.

Here, we identified a novel *MMP21* variant in one Han individual from among 78 patients with dextrocardia by WES.

## Case Presentation

Seventy-eight patients were recruited in this study from Second Xiangya Hospital after providing written informed consent, and family history was extensively investigated. After strict clinical and radiographic examination, all patients were diagnosed with dextrocardia with or without primary ciliary dyskinesia (PCD) and/or CHD. This study was approved by the Ethics Committee of the Second Xiangya Hospital of Central South University.

**Figure 1:**
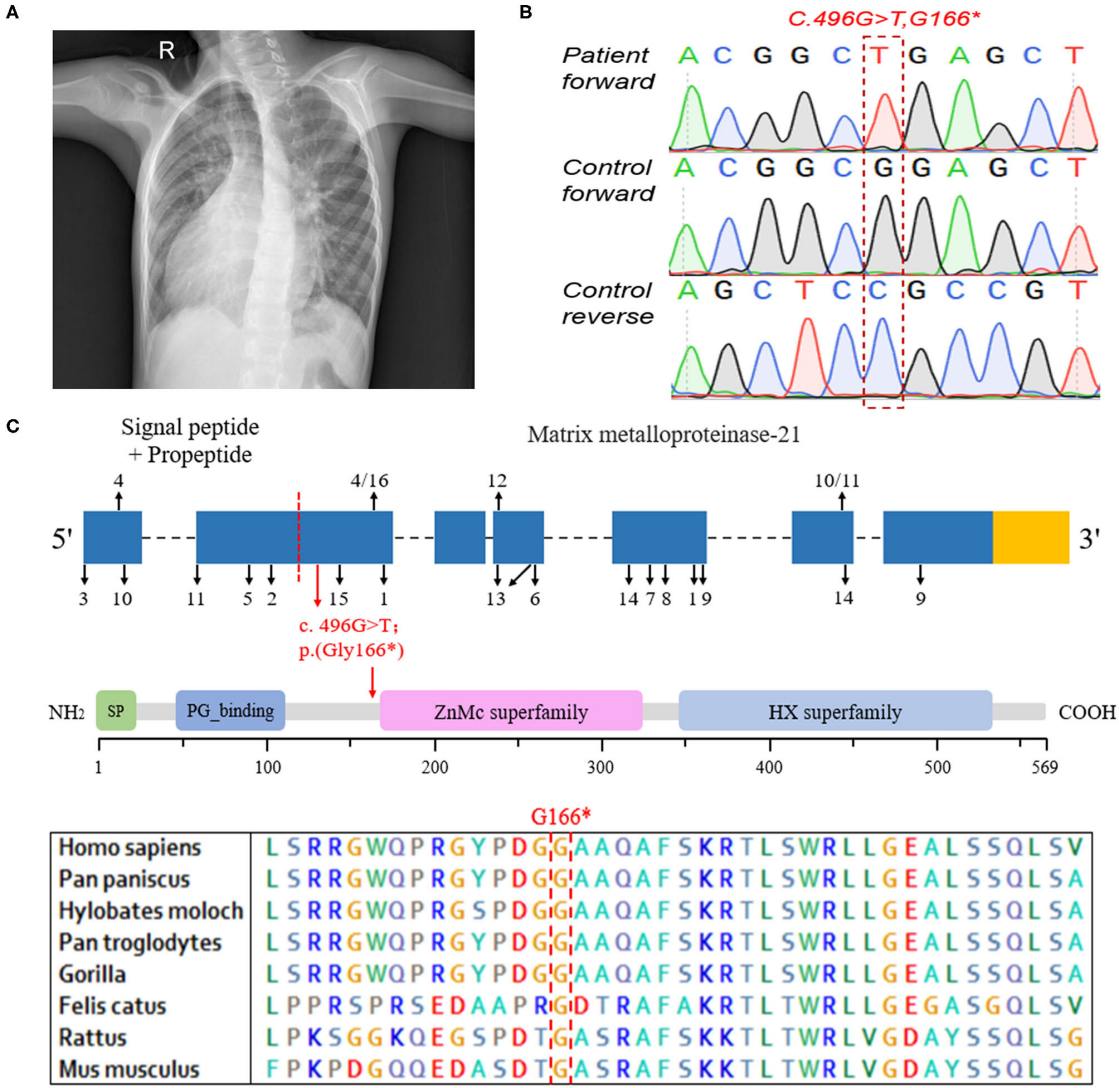
The clinical data and genetic analysis of the patient with *MMP21* variant. **(A)** Chest X-ray of the patient indicating dextrocardia. **(B)** Sanger DNA sequencing chromatogram demonstrating the homozygosity for an *MMP21* stopgain variant (c.G496T; p.G166*). **(C)** The sequence encoding the signal peptide and propeptide is before the red dotted line, the subsequent sequence encodes matrix metalloproteinase-21, the red arrow indicates the variant identified in this study, and the numbers and black arrows indicate the family in Table 2 and position of *MMP21* variants, respectively. Domain structure of MMP21. SP, signal peptide; PG_binding, putative peptidoglycan-binding domain; ZnMc superfamily, zinc-dependent metalloprotease; HX superfamily, hemopexin-like repeats.

The patient with MMP21 variation, a 7-year-old boy, was born at full term and liable to catch colds but developed normally. He was diagnosed with complex CHD, mirror-image dextrocardia (Figure 1A), single-ventricle, pulmonary stenosis, and transposition of the great arteries, without PCD. According to the parents of the proband, there are no other people with cardiovascular diseases in his family. A total cavopulmonary connection operation was conducted for the patient and achieved a good outcome. Written informed consent was obtained from the legal guardians of the patient for the publication of any potentially identifiable images or data included in this article.

### Genetic Analysis

Whole peripheral blood samples of the patients were obtained and stored in EDTA tubes. Genomic DNA was extracted by QIAamp DNA Blood Mini Kit (250) (Qiagen, Valencia, CA, USA).

The WES was mainly performed in the Novogene Bioinformatics Institute (Beijing, China). The exomes were captured by means of Agilent SureSelect Human All Exon V6 kits and sequenced on Illumina NovaSeq6000 (Illumina Inc, San Diego, USA). The WES data was filtered using three criteria: (1) Variations outside the coding regions (e.g., intergenic, intronic, and untranslated regions) and synonymous variants were excluded; (2) high allele frequency (>0.01%) compared to population-based databases (e.g., 1,000 Genomes Project, ESP, ExAC, and gnomAD) were excluded; and (3) prediction of a deleterious functional effect using bioinformatics programs (e.g., MutationTaster, CADD, SIFT, and Polyphen2), loss of function, and damaging variations were reserved.

Sanger sequencing was performed to validate the identified variant, and the primers were designed by PrimerQuest Tool (F: 5′-TTTCTCCCTGTCTGTvCTCTCT, R: 5′-GTGCTCTTACCTCTCCCAAAG). PCR conditions consisted of 94°C for 30 s, 55°C for 30 s, and 72°C for 1 min, for a total of 35 cycles using 2 × Power Taq PCR MasterMix (BioTeke, Beijing, China). PCR products were electrophoresed on 1% agarose gels. The PCR fragment was subsequently cut, and purified fragments were sequenced on 3730XL sequencer (Applied Biosystems).

### Identification of MMP21 Variation

WES was conducted and generated 12 Gb data with 99% coverage and a depth of >100 ×. After filtration of the WES data (Table 1), we finally identified a biallelic stopgain variant (c.G496T; p.G166^*^) in *MMP21* and further confirmed it by Sanger sequencing (Figure 1B). This variant caused a truncated MMP21 mRNA containing only the signal peptide and propeptide, while the coding sequence of matrix metalloproteinase-21 was almost entirely absent (Figure 1C). This variant was predicted to be “disease causing” by MutationTaster and not found in the 1,000 Genome Browser, the ExAC Browser, the Exome Variant Server, or 200 unrelated ethnically matched healthy controls. The controls were individuals presenting for routine health checkups or volunteers without similar symptoms or any positive family history of cardiovascular disorders (male/female: 100/100, mean age 36.1 ± 4.3 years). In addition, the variant had the highest CADD score of 35 suggesting that it is deleterious. Multiple alignment of mmp21 orthologs in other animal species showed that the amino acid sequence after position 166 is highly conserved. According to ACMG standards and guidelines, this variant was categorized as pathogenic (PVS1, PM2, PM4, PM6, and PP3) and identified as the genetic lesion of the patient.

**Table 1:** The gene list after WES data filtration of the patient.

**CHR**	**POS**	**REF**	**ALT**	**GeneName**	**Mutation**	**Genotype**	**OMIM**	**Database**	**MutationTaster**	**CADD**	**ACMG**	
17	56,598,212	AG	A	SEPTIN4	NM_001198713: exon11:c. 1254–10C>-	Hom	–	Unknown variant	0.99999,D	–	PM2,PM6	Uncertain significance
10	5,032,240	G	GGA	AKR1C2	NM_001354: exon11:c. 930–10–>TC	Hom	AR; 46XY sex reversal	Known variant	0.99999,P	−0.170684, 3.168	BP4	Uncertain significance
X	152,988,952	TG	T	BCAP31	NM_001139457: exon1:c.157 +10C>-	Hom	AR; Deafness, dystonia, and cerebral hypomyelination	Unknown variant	0.99976,P	–	PM2, PM6	Uncertain significance
X	135,574,521	AG	A	BRS3	NM_001727: exon3:c.1188 delG:p.E396fs	Hom	–	Known variant	0.99999,P	–	PM4	Uncertain significance
X	103,267,971	C	G	H2BFWT	NM_001002916: exon1:c.G26 2C:p.V88L	Hom	–	Known variant	0.99906,D	2.543224, 19.74	PM2,PP3	Uncertain significance
6	33,037,413	C	CA	HLA-DPA1	NM_001242524: exon3:c.346+5->T	Hom	–	Unknown variant	0.99999,P	–	PM2, PM6	Uncertain significance
10	127,462,601	C	A	MMP21	NM_147191: exon2:c.G49 6T:p.G166X	Hom	AR; Heterotaxy	Unknown variant	1,D	9.265177, 35	PVS1,PM2, PM4,PM6,PP3	Pathogenic
13	24,798,487	C	T	SPATA13	NM_001166271: exon2:c.C142 0T:p.P474S	Hom	–	Known variant	0.99717,P	4.289395, 24.0	–	Uncertain significance

*CHR, Chromosome; POS, position; REF, reference sequence base; ALT, alternative base identified; Hom, homozygous; AR, autosomal recessive; P, polymorphism; D, disease causing; BP, Benign Supporting; PP, Pathogenicity Supporting; PM, Pathogenicity Moderate; PVS, Pathogenicity Very Strong. The database included 1000G, ESP and gnomAD browser*.

## Discussion

Our group aimed to identify laterality defect genes in a cohort of 78 patients with dextrocardia and CHD. Approximately 10% had variations in genes, i.e., *NODAL, ZIC3, NKX2-5*, and *CCDC151*. WES was conducted to identify the causative genes in these patients. A novel biallelic stopgain *MMP21* variant (c.G496T; p.G166^*^) was identified and predicted to be a disease-causing variant by MutationTaster and Combined Annotation Dependent Depletion (CADD). This variant was also not present in the current 1,000 Genome Browser, the ExAC Browser, or the Exome Variant Server. This variant caused a truncated MMP21 mRNA containing only a signal peptide and propeptide, while two functional domains (ZnMc superfamily; HX superfamily) were entirely absent, which may induce the decay of MMP21 mRNA in the patient according to nonsense-mediated mRNA decay theory (12, 13). *MMP21* variants that caused laterality defects and complex CHD in humans are summarized in Table 2. To our knowledge, this novel variant (c.G496T; p.G166^*^) was the first homozygous stopgain variant identified in dextrocardia patients and the first *MMP21* variant found in mainland China, suggesting the critical role of *MMP21* during left–right patterning in the Han Chinese population.

**Table 2:** The summary of variants in *MMP21*.

**Family**	**Cardiac anomalies**	**Extra-cardiac laterality defects**	**Mutations**	**Protein change**	**Inheritance**	**Reference**
1	Complex CHD, dextrocardia	Intestinal malrotation, polysplenia	c.677T>C, c.1203G>A	p.(Ile226Thr), p.(Trp401*)	Compound heterozygous	(9)
2	Complex CHD, dextrocardia	Situs ambiguus (spleen, liver)	deletion of exons 1-3, c.365delT	p.(Met122Serfs*55)	Compound heterozygous	
3	Complex CHD	Midline liver, intestinal malrotation, polysplenia	c.1A>G	p.(Met1?)	Homozygous	
4	Complex CHD	Left pulmonary isomerism, left sided liver, right-sided stomach, polysplenia.	c.91C>T, c.643G>A	p.(Arg31Trp), p.(Glu215Lys)	Compound heterozygous	
5	CHD, dextrocardia	Situs ambiguus (thoracic and abdominal)	c.308_309delAG	p.(Glu103Alafs*154)	Homozygous	
6	Complex CHD	Situs ambiguus (thoracic) midline liver, intestinal malrotation	c.961G>C	p.(Ala321Pro)	Homozygous	
7	Complex CHD	Situs ambiguus (thoracic and abdominal)	c.1078C>T	p.(Arg360Cys)	Homozygous	
8	Complex CHD	Situs ambiguus (abdominal) or Situs inversus totalis	c.1124G>A	p.(Arg375His)	Homozygous	
9	CHD, dextrocardia	None	c.1222C>G, c.1585_1588dup	p.(Arg408Gly), p.(Val530Glyfs*3)	Compound heterozygous	
10	Complex CHD	Thoracic situs inversus	c.101C>T, c.1372C>T	p.(Ser34Leu), p.(Arg458*)	Compound heterozygous	
11	Complex CHD, dextrocardia	None	c.163C>T, c.1372C>T	p.(Arg55Trp), p.(Arg458*)	Compound heterozygous	
12	Complex CHD, dextrocardia	None	c.1024_1025delAA	p.(Lys342Argfs*13)	Homozygous	(10)
13	Complex CHD, dextrocardia	Right-sided stomach	c.847C>T, c.947G>A	p.(His283Tyr), p.(Trp316*)	Compound heterozygous	(8)
14	Complex CHD, dextrocardia	None	c.1380_1381delGA, c.854T>C	p.(Lys461Valfs*14), p.(Ile285Thr)	Compound heterozygous	
15	Situs anomaly		c.557G>T	p.(Ser186Ile)	Homozygous	(11)
16	Situs anomaly		c.643G>A	p.(Glu215Lys)	Homozygous	
17	Complex CHD, dextrocardia	None	c. 496G>T	p.(Gly166*)	Homozygous	Present study

The known pathogenic genes of laterality defects account for only 15–20% of cases and are mainly related to the NODAL/TGFβ signaling pathway, SHH signaling pathway, and ciliary functions (11). Nodal cilia play a role in the origination of L–R asymmetry patterning (14–17). The observation that more than half of patients with primary ciliary dyskinesia (PCD) accompanied by SIT or HTX further reinforces the connection (2). In a prospective study involving 767 participants, Shapiro et al. (2) found that at least 12.1% of patients with classical PCD have HTX ranging from classic to subtle. In our previous studies, we reported a novel *CCDC151* mutation in a patient with PCD and SIT and computed tomographic scanning of the sinus and lungs showing diffuse bronchiectasis and chronic sinusitis, respectively. His nasal nitric oxide concentrations (nNO) were far below (2 ppb) the PCD-specific nNO cutoff value (287 ppb). These results indicated that the *CCDC151* mutation damaged cilia and further affected L–R patterning (18). However, we did not find PCD or other clinical phenotypes related to cilia in the *MMP21* variant patient, suggesting that *MMP21* was unrelated to cilia and involved in L–R patterning in another way. In addition, some known variants of *NODAL, ZIC3*, and *NKX2-5* were identified in this cohort.

Knockdown or genome editing of the *MMP21* ortholog in zebrafish resulted in heart-looping defects in a dose-dependent manner (9, 10). Mutant zebrafish embryos also showed concomitant disruption of the laterality marker southpaw in the lateral plate mesoderm and disrupted notch signaling *in vitro* and *in vivo*, suggesting that the heart-looping defect is related to abnormal L–R patterning and that *MMP21* is a negative regulator of notch signaling (10). Consistent with the findings from zebrafish, mutant mouse embryos generated by Mmp21 genome editing and N-ethyl-N-nitrosourea (ENU)-induced mice that have a homozygous missense mutation in Mmp21 both exhibit CHD and laterality defects (7). The findings suggested that *MMP21* plays an important role in the establishment of asymmetric organ development.

The Drosophila genome encodes two Mmps, Mmp1 and Mmp2. Experimental results from Drosophila demonstrated that Mmps play essential roles in promoting ECM remodeling, cell polarization, and lumen formation during Drosophila cardiogenesis. In humans, up to 25 Mmps have been identified with overlapping functions, and only *MMP21* has been confirmed to be involved in L–R patterning (5, 19). It is very significant to identify the deleterious variants affecting L–R patterning in other members of the MMP family in both human and animal models.

In conclusion, our finding expands the spectrum of *MMP21* variants and provides extra support that *MMP21* play important roles in L–R patterning.

## Data Availability Statement

The datasets generated for this study can be found in NCBI SRA, NCBI Accession No. PRJNA668249.

## Ethics Statement

The studies involving human participants were reviewed and approved by Ethics Committee of the Second Xiangya Hospital of the Central South University. A signed written informed consent was obtained from the legal guardians of the patient for the publication of any potentially identifiable images or data included in this article.

## Author Contributions

Z-PT designed the overall study and performed data analysis. Z-ZY and L-LF processed the WES data, validated the mutation, and drafted the manuscript. Z-CJ contributed to the study as summer intern. Y-FY enrolled the patients. All authors read and approved the final version of the manuscript. All authors contributed to the article and approved the submitted version.

## Conflict of Interest

The authors declare that the research was conducted in the absence of any commercial or financial relationships that could be construed as a potential conflict of interest.
